# Safety and Efficacy Assessment of Two New Leprosy Skin Test Antigens: Randomized Double Blind Clinical Study

**DOI:** 10.1371/journal.pntd.0002811

**Published:** 2014-05-29

**Authors:** Becky L. Rivoire, Nathan A. Groathouse, Stephen TerLouw, Kapil Dev Neupane, Chaman Ranjit, Bishwa Raj Sapkota, Saraswoti Khadge, Chatra B. Kunwar, Murdo Macdonald, Rachel Hawksworth, Min B. Thapa, Deanna A. Hagge, Melinda Tibbals, Carol Smith, Tina Dube, Dewei She, Mark Wolff, Eric Zhou, Mamodikoe Makhene, Robin Mason, Christine Sizemore, Patrick J. Brennan

**Affiliations:** 1 Department of Microbiology, Immunology, and Pathology, Colorado State University, Fort Collins, Colorado, United States of America; 2 Mycobacterial Research Laboratory, Anandaban Hospital, Kathmandu, Nepal; 3 The EMMES Corporation, Rockville, Maryland, United States of America; 4 Division of Microbiology and Infectious Diseases, National Institute of Allergy and Infectious Diseases, National Institutes of Health (NIH), Bethesda, Maryland, United States of America; University of California San Diego School of Medicine, United States of America

## Abstract

**Background:**

New tools are required for the diagnosis of pre-symptomatic leprosy towards further reduction of disease burden and its associated reactions. To address this need, two new skin test antigens were developed to assess safety and efficacy in human trials.

**Methods:**

A Phase I safety trial was first conducted in a non-endemic region for leprosy (U.S.A.). Healthy non-exposed subjects (n = 10) received three titrated doses (2.5 µg, 1.0 µg and 0.1 µg) of MLSA-LAM (n = 5) or MLCwA (n = 5) and control antigens [Rees MLSA (1.0 µg) and saline]. A randomized double blind Phase II safety and efficacy trial followed in an endemic region for leprosy (Nepal), but involved only the 1.0 µg (high dose) and 0.1 µg (low dose) of each antigen; Tuberculin PPD served as a control antigen. This Phase II safety and efficacy trial consisted of three Stages: Stage A and B studies were an expansion of Phase I involving 10 and 90 subjects respectively, and Stage C was then conducted in two parts (high dose and low dose), each enrolling 80 participants: 20 borderline lepromatous/lepromatous (BL/LL) leprosy patients, 20 borderline tuberculoid/tuberculoid (BT/TT) leprosy patients, 20 household contacts of leprosy patients (HC), and 20 tuberculosis (TB) patients. The primary outcome measure for the skin test was delayed type hypersensitivity induration.

**Findings:**

In the small Phase I safety trial, reactions were primarily against the 2.5 µg dose of both antigens and Rees control antigen, which were then excluded from subsequent studies. In the Phase II, Stage A/B ramped-up safety study, 26% of subjects (13 of 50) showed induration against the high dose of each antigen, and 4% (2 of 50) reacted to the low dose of MLSA-LAM. Phase II, Stage C safety and initial efficacy trial showed that both antigens at the low dose exhibited low sensitivity at 20% and 25% in BT/TT leprosy patients, but high specificity at 100% and 95% compared to TB patients. The high dose of both antigens showed lower specificity (70% and 60%) and sensitivity (10% and 15%). BL/LL leprosy patients were anergic to the leprosy antigens.

**Interpretation:**

MLSA-LAM and MLCwA at both high (1.0 µg) and low (0.1 µg) doses were found to be safe for use in humans without known exposure to leprosy and in target populations. At a sensitivity rate of 20–25% these antigens are not suitable as a skin test for the detection of the early stages of leprosy infection; however, the degree of specificity is impressive given the presence of cross-reactive antigens in these complex native *M. leprae* preparations.

**Trial Registration:**

ClinicalTrails.gov NCT01920750 (Phase I), NCT00128193 (Phase II)

## Author Summary

Clinically useful skin test reagents should be safe and sufficiently sensitive to detect infection prior to physical manifestations of leprosy disease. While in these small scale human studies, leprosy reagents were safe for use in humans, they failed in respect of sensitivity at a rate of 20–25% in the key indicator group, BT/TT leprosy patients. Specificity in terms of leprosy vs. tuberculosis at a rate of 95–100% was surprisingly high in light of the extensive presence of cross-reactive antigens in the complex native *M. leprae* preparations. These results could justify a further trial at lower dosages.

## Introduction

Despite the success of the widely applied multidrug therapy (MDT) in reducing the prevalence of leprosy, transmission continues with new case detection maintaining a steady rate in endemic regions of high burden areas, further emphasizing the importance of early detection [1]. A sensitive and specific test would facilitate early detection, allowing treatment prior to manifestation of physical disease, and reduced transmission, reactions, and patient disabilities. Such a test should be sensitive to the earliest immunological response to *M. leprae*, inexpensive for use in leprosy endemic regions, and simple to implement. Intradermal skin testing through measurement of a delayed type hypersensitivity (DTH) reaction could meet the sensitivity attributes, and does so with Tuberculin DTH in the context of tuberculosis. [2], [3] Earlier skin test antigens for leprosy (Lepromin A, Rees Antigen, and Convit's antigen) [4], [5] proved safe when used in humans. Lepromin A has been used for nearly 40 years [6], [7] and provides a solid foundation of safety. In the present context, two new skin test antigens, *Mycobacterium leprae* soluble antigens (MLSA) devoid of glycolipids particularly lipoarabinomannan (LAM) called MLSA-LAM and MLCwA (*M. leprae* cell wall associated antigens), were generated, reflective of those of Convit and Rees in that they were derived from *M. leprae* grown in armadillos, but prepared under rigorous current good manufacturing practices (GMP) [8], [9]. The primary goal of the application of these antigens in a clinical trial was to determine if the products were well tolerated in humans and if they exhibited diagnostic potential that would justify larger trials and eventual clinical implementation.

## Methods

### Ethics Statement

The Colorado State University (CSU) Institutional Review Board (IRB) for the protection of human subjects and the Nepal Health Research Council (NHRC) approved the protocols and informed consent forms, and certified that the studies were ethically cleared to proceed. All subjects provided written informed consent; no children were enrolled in the study. The Phase I safety non-endemic trial (registration number: NCT01920750) and Phase II safety and efficacy endemic trial (registration number: NCT00128193) were registered with ClinicalTrials.gov. The Phase I trial was not registered prior to implementation, because the trial was completed (February, 1999), before ClinicalTrials.gov registry was made available to the public (February, 2000). Retrospective registration of the Phase I trial was requested and obtained for publication by PLoS Tropical Diseases.

### Intervention and Control Products

Test antigens were MLSA-LAM and MLCwA at 2.5 µg, 1.0 µg, and 0.1 µg/0.1 ml dose [10]. Control antigens were Rees MLSA [11], 20×1.0 ml vials at 1.0 µg dose received as a gift from Philip Draper and the late Joseph Colston (National Institute Medical Research, Mill Hill, UK) in Phase I; 0.9% sterile saline, approved for human use (Abbott Laboratories Inc., Abbott Park, IL) in Phase I and Phase II, Stage A; Tubersol Tuberculin PPD, 5 TU dose (Aventis Pasteur Inc., Swiftwater, PA) in Phase II Stage A and B and preliminary testing of Stage C-1a; and, Tuberculin PPD RT 23, 2 TU dose, solution for injection (Statens Serum Institute (SSI), Copenhagen, Denmark) in the remainder of Stage C-1 a/b. Strong reactions to the 5 TU dose of PPD prompted unblinding and SMC review for this one product. The outcome was a recommendation from the sponsor to use a lower dose for the remainder of Stage C-1 studies; this change was not expected to significantly impact the study results. The lower dose was not available from the same vendor; therefore, a new vendor was used.

### Study Design

The Phase I clinical trial was conducted on MLSA-LAM and MLCwA in a non-endemic region for leprosy, while the Phase II clinical trial was conducted on both antigens in an endemic region for leprosy. The Phase II clinical trial included compulsory testing for safety and efficacy in healthy subjects without known exposure to leprosy and in target populations: borderline tuberculoid/tuberculoid (BT/TT) and borderline lepromatous/lepromatous (BL/LL) leprosy patients, household contacts of BL/LL leprosy patients (HC), and tuberculosis patients (TB).

Prior to initiation, the protocol was amended: 1) to allow testing in smaller group sizes (n = 20) to assure safety before ramping and to improve the likelihood of recruiting the requisite number of subjects; 2) to decrease the number of injections for subject comfort; and, 3) to add comparative *in vitro* assays to maximize the potential of this study (unpublished work). Stage C was divided into Stage C-1 for small scale studies and Stage C-2 for ramping to achieve statistical significance. Stage C-2 was not feasible, due to cessation of dedicated funding. Stage C-1 was divided into two parts, a and b, to test the high dose (1 µg) and low dose (0.1 µg) of each antigen. This protocol change enabled both the reduction of sample size and number of injections per subject, while remaining within the scope of the original protocol.

Participants in the Phase I safety trial (U.S.A.) were between the ages of 18 and 40 years, with a weight greater than 100 lbs (45 kg) for females and 140 lbs (64 kg) for males. In the Phase II trial, there were 68% male and 32% females enrolled in the study. The mean age of the subjects was 29 y, with a range of 18 to 60 y. Age did not vary significantly across study groups in Stages A, B, and C-1b, but there was a significant difference in age across study groups in Stage C-1a, p<0.05. Phase II participants had a weight greater than 30 kg for females and 38 kg for males.

The method for antigen administration and measuring induration was adapted from “Guidelines for Conducting Skin Test Surveys in High Prevalence Countries,” issued by the International Union against Tuberculosis and Lung Disease [12]. In the Phase I clinical trial, each participant received five 100 µl intradermal injections of titrated doses (2.5 µg, 1.0 µg, and 0.1 µg) of one of the two skin test antigens, one injection of 0.9% sodium chloride, and one injection of Rees MLSA control leprosy skin test antigen at a 1.0 µg dose, between both forearms. Participants in the Phase II, Stage A/B clinical trial, each received four injections of titrated doses (1.0 µg and 0.1 µg) of one of the two skin test antigens, one injection of 0.9% sterile saline (Stage A only), and one injection of Tuberculin PPD Tubersol 5 TU, between both forearms. Stage C-1a/b subjects received three injections of the high dose (1.0 µg) or low dose (0.1 µg) of each intervention and control antigen.

DTH responses were read at ∼15 min, 48 h and 72 h post injection in the Phase I and Phase II, Stage A studies; prior to Stage B, the 48 h reading was dropped and a 7 day reading was added for convenience and safety. The 15 min observation was primarily a safety measure to gauge for immediate adverse events, such as anaphylaxis and a 30 min observation was added to the Stage C-1 studies to assure that subjects were not adversely affected from an added blood draw for *in vitro* testing (unpublished work). If a subject was observed to have an induration greater than 10 mm at any injection site, at either subsequent study visit, he/she was asked to return at 25–31 days for a final induration measurement. Any persistent reaction was followed-up until resolved or stabilized.

Each antigen site was evaluated for reactogenicity, defined as a reaction at the site of injection that is common and reasonably expected for the intervention being studied. Specifically, the maximal diameter of induration and erythema, and presence of pain, pruritus (itching), bleeding, urticaria (hives), infection, or blistering were possible reactions based on the history of Tuberculin skin testing [12], [13]. Classification of reactogenicity by grade was outlined in the Clinical Study Reactogenicity Assessment Table included in the Phase II Clinical Protocol. Reactions were graded as mild (1), moderate (2), severe (3), or life-threatening (4). Severe reactions were recorded as adverse events, while life-threatening reactions were recorded as severe adverse events. Adverse events (AEs) were coded by the Medical Dictionary for Regulatory Activities (MedDRA) [14] for preferred term and system organ class (SOC).

The Safety Monitoring Committee (SMC) [15] for the Phase I study consisted of two off-site leprologist physicians. The Phase II, Stage A/B study SMC consisted of 4 physicians: two off-site leprologists, one off-site infectious disease doctor, and one on-site tuberculosis doctor who served as the independent safety monitor (ISM).

### Phase I Safety Trial - Non-Endemic U.S.A

Phase I study commencement was December 1998 and study completion was February 1999. The total sample size of 10 subjects was divided between two antigen groups; 5 subjects received titrated doses of MLSA-LAM or MLCwA, plus control antigens. Healthy subjects without any known contact with tuberculosis or leprosy patients were recruited from the student body at CSU. All subjects were Tuberculin skin test negative when tested 3 weeks prior to study initiation. Subjects were assigned to either the MLSA-LAM or MLCwA antigen group based on a random sequence of integers. Study objectives were two-fold: to determine if MLSA-LAM and MLCwA were safe to use in humans as skin test antigens; and, to determine that the range of concentrations chosen for skin testing did not elicit a reactive response in a negative control group of human subjects from a region non-endemic for leprosy. The expected outcome for the Phase I clinical trial was that all three concentrations of the two leprosy skin test antigens, saline, and control Rees MLSA leprosy antigen would not evoke a skin test antigen response. Any untoward local reaction, such as severe erythema or necrosis, would result in those antigen doses being dropped from further testing.

### Phase II Stage A/B Safety Trial - Leprosy Endemic Area in Nepal

The Phase II, Stage A study commenced in April 2002 and was completed in July 2002; Stage B study commenced in May 2003 and was completed in January 2004. The Phase II, Stage A and B clinical study was similar in design to the Phase I clinical trial, in that 100 subjects were divided between two groups; 50 subjects received titrated doses of MLSA-LAM and MLCwA, plus control antigens. Stage A was a preliminary safety screen with 10 subjects from a leprosy endemic region, whereas Stage B completed the safety trial with 90 subjects. Healthy subjects without any known contact with tuberculosis or leprosy patients were recruited from the Lalitpur Nursing Campus, Sanepa, Kathmandu and the Dhulikel Medical Institute, Dhulikel, Nepal, following delivery of a recruitment talk by a senior member of the research team from Anandaban Hospital, Kathmandu, Nepal, using the local Nepali language or English with immediate translation to Nepali.

To assess eligibility, volunteers were asked a series of health related questions, given a general physical exam and standard examination for signs of leprosy [16], [17]. Females who were pregnant or lactating or individuals who were on corticosteroid or other immunosuppressive treatment, had cancer, diabetes, known hypersensitivities or allergies, expatriates other than those from India, had participated in an earlier Stage of this study, or were concurrently participating in another clinical trial were excluded from this study. Demographic information was collected, and BCG scar measured across the largest diameter (if present). Familial relationships were recorded for HC of leprosy patients only.

Phase II subjects were assigned an antigen and administration template based on a fixed block randomization sequence provided by the Data Coordinating Center (DCC; The EMMES Corporation, Rockville, MD). Antigens were concealed by antigen codes randomized for each antigen and antigen dose; both randomization schemes were sent to the clinical study principal investigator in the event that unblinding was necessary. Antigen codes were provided in separate envelopes, such that if only one antigen required unblinding, the others were not compromised.

The Phase II, Stage A and B clinical trials were performed by staff from Anandaban Hospital at the two sites. Study objectives were to evaluate the safety and to select a dose of MLSA-LAM and MLCwA causing minimal induration in healthy subjects without known exposure to clinical leprosy or tuberculosis that are living in a region endemic for leprosy. Subjects in the Phase II, Stages A and B clinical trials were expected to have a small (less than 10 mm) induration reaction to the leprosy skin test antigens and PPD, due to expected extensive cross-reactivity to *M. tuberculosis*, BCG vaccination, and/or environmental mycobacteria [18]–[20].

### Phase II, Stage C Trial for Safety, Specificity, and Sensitivity - Leprosy Endemic Region

The Phase II, Stage C-1a study commenced in December 2006 and was completed in March 2008; Stage C-1b study lasted from May 2009 to September 2009. Two protocol amendments were filed, one in May 2007 to decrease the control antigen PPD dose; and, one in March 2009 to reduce the study size and to add comparative *in vitro* tests. The Phase II, Stage C-1a involved a high dose (1.0 µg) group (n = 80) and Stage C-1b a low dose (0.1 µg) group (n = 80). Each included 20 BT/TT leprosy patients, 20 BL/LL leprosy patients, 20 HC of BL/LL leprosy patients, and 20 TB patients. Leprosy patients had one or more of the cardinal signs of leprosy including: hypopigmented or erythematous skin lesion(s) with loss of sensation; thickened peripheral nerves; or positive acid-fast bacilli (AFB) in slit skin smears or biopsy material [21]. Subjects with leprosy were either receiving or had completed standard MDT treatment for leprosy, no more than four years prior to study enrollment. Household contacts were determined to be healthy by history and physical examination and had resided in the same residence as the BL/LL leprosy index case for at least 6 months duration and within 6 months of this study. Tuberculosis patients had either at least two initial sputum smear examinations positive for AFB, or one sputum examination positive for AFB and radiographic abnormalities consistent with pulmonary tuberculosis, or one sputum specimen positive for AFB and culture positive for AFB. All patients had completed the intensive phase of chemotherapy for tuberculosis, but were still undergoing the continuation phase of therapy [22].

Leprosy patients and their household contacts were recruited at Anandaban Hospital, Kathmandu, and tuberculosis patients from the Tuberculosis Clinic of Patan Hospital, Kathmandu. Explanation of the study was guided by use of an appropriate flip chart and consent form translated into the native language, either Nepali or Hindi. In the case of illiterate subjects, information was read to them by a staff member. Randomization and blinding was the same as described for Phase II, Stage A/B.

Phase II, Stage C-1 studies were performed at the recruitment site by staff from Anandaban Hospital. A total of 10–12 ml of blood was collected from each participant prior to antigen administration for *in-vitro* laboratory testing involving quantitation of the release of IFNγ from lymphocytes after stimulation in whole blood and standard serology based on the phenolic glycolipid-I (PGL-I) antigen (unpublished work). Remaining subject samples were destroyed at the end of the study, per NHRC request.

The primary objective of the Phase II, Stage C-1 study was the assessment of safety in target populations: BT/TT and BL/LL leprosy patients, HC of BL/LL leprosy patients, and TB patients. The secondary objective was the assessment of efficacy of the two skin test antigens by comparing induration measurements following skin test administration. It was expected that BT/TT leprosy patients and some healthy contacts of leprosy patients would have large indurations at *M. leprae*-derived antigen sites; BL/LL leprosy patients would have negative indurations at all leprosy skin test sites due to *M. leprae* specific T-cell anergy; and, TB patients would react with a large induration at the PPD site and may react with an induration less than 10 mm at the leprosy antigen sites.

### Statistical Analysis

For the Phase I clinical study, both antigens at each dose were not expected to elicit a DTH skin test response; therefore, a sample size of 10 subjects (5 per group) was expected to be satisfactory as a preliminary safety screen in a non-endemic region for leprosy. For the Phase II, Stage A clinical study, both antigens and antigen doses were expected to show minimal reactions, if any, and therefore a sample size of 10 subjects was expected to uncover any major safety concerns. For the Phase II, Stage B clinical study, the sample size was increased by 40 subjects for each antigen, to generate statistically significant data. Sample size consideration analysis indicated that the study would be able to meet the primary statistical objectives should up to 10% of the subjects be lost to follow-up. The Phase II, Stage C-1a/b trial was designed to assess the safety and primary response measure of induration resulting from skin test antigen DTH responses in small numbers (n = 20) of participants within each of four different groups that theoretically may be at higher risk of serious adverse responses to novel antigens. The probability of observing one or more serious adverse events related to antigen administration was calculated. If the true serious adverse event rate is 10%, then there is an 85% chance of observing one or more serious adverse events in any one of the four groups with loss during follow-up of 10% of the subjects, or 88% if there is no loss during follow-up. Kruskal-Wallis tests were used to compare age by study group [23]. Efficacy analyses were performed using the receiver operating characteristic (ROC) curve [24]. Graph Pad, Prism for Windows, version 6.0 (La Jolla, CA) was used for graphing and analyzing ROC curves.

## Results

**Figure 1:**
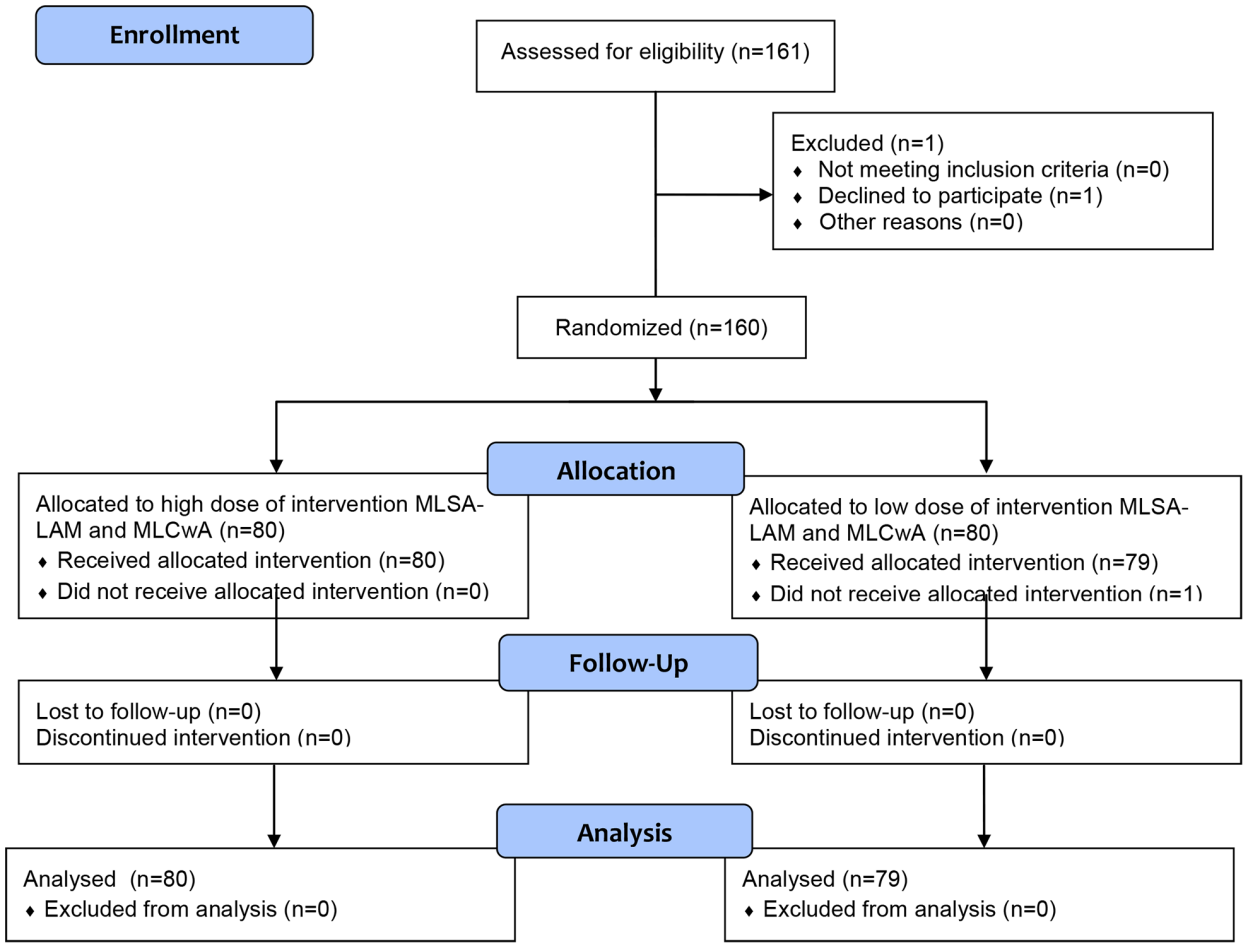
CONSORT flow diagram, Phase II, Stage C-1 Trial.

In the Phase I trial, eleven volunteers were recruited. Ten volunteers met the inclusion criteria and were enrolled in the study; one volunteer was unable to participate. One hundred and one volunteers were recruited for Stage A and B and one declined participation. The Phase II, Stage C-1a/b Trial CONSORT Flow Diagram is shown in **Figure 1**. Over two subsequent studies, one-hundred and sixty-one subjects (81 for Stage C-1a and 80 for Stage C-1b) were recruited; one HC in Stage C-1a declined participation and one BL/LL leprosy patient in Stage C-1b declined antigen administration.

Classification of leprosy patients in Stage C-1a were 20 of 20 (100%) BT subjects in the BT/TT group, and 12 of 20 (60%) BL and 8 of 20 (40%) LL subjects in the BL/LL group. Stage C-1b leprosy patients consisted of 17 of 20 (85%) BT and 3 of 20 (15%) TT subjects in the BT/TT group, and 14 of 20 (70%) BL and 6 of 20 (30%) LL in the BL/LL group. All but nine subjects were treated with multibacillary MDT treatment, the others received paucibacillary MDT [25]. A total of 41 of 80 (51%) subjects between the two Stages had completed their treatment. None of the leprosy patients had a record of being skin tested with Lepromin A. History of type 1 leprosy reaction [26] in Stage C-1a was 6 of 20 (30%) for BT/TT and 5 of 20 (25%) for BL/LL subjects; and, Stage C-1b was 3 of 20 (15%) for BT/TT and 5 of 20 (25%) for BL/LL subjects. Erythema nodosum leprosum (ENL) reaction [27] history in Stage C-1 a/b were recorded for 3 of 20 (15%) and 4 of 20 (20%) in BL/LL leprosy subjects.

### Protocol Deviations

There were no protocol deviations that are believed to have affected product stability or resulted in adverse events. Deviations did not occur during the Phase I trial and Phase II, Stage A study. In the Phase II, Stages B and C-1 studies, the most frequent deviations were due to convenience of participant, and subject unable to comply.

### Choice of Control Antigens

The Rees antigen (MLSA) served as the reference antigen for the small safety Phase I study conducted at CSU, but the NIH regulatory authority would not allow use in the safety and efficacy Phase II studies conducted in Nepal, because it was not commercially available or registered under a U.S.A. Investigational New Drug (IND) [28]. Lepromin A, prepared at the National Hansen's Disease Program, Baton Rouge, LA, for WHO was no longer available and existing present day Lepromin A is prepared in India and Cuba, and not approved for U.S.A. studies. Tuberculin PPD, the skin test antigen for tuberculosis, was therefore used.

### Dosages

The dose range of leprosy antigens (2.5 µg, 1.0 µg, and 0.1 µg) was chosen based on the Rees antigen dose of 1.0 µg [29], potency studies in sensitized guinea pigs [30], and antigen availability. The low dose (0.1 µg) was based on the lowest concentration of Rees antigen tested in the field [29], [31], and this was the limit of detection for DTH responses in guinea pigs sensitized with *M.* leprae [10]. The standard Tuberculin test dose is equivalent to 5 TU of PPD-S, defined as the delayed skin test activation contained in a 0.1 µg/0.1 ml dose of PPD-S [32]. This dose is based on dry weight, whereas the leprosy dosage is based on measured protein content.

### Safety

Safety was analyzed by reactogenicity and frequency, severity, and relationship of adverse events to the investigational product. Observations were tabulated by maximum number of events across all readings by antigen, except the 15 min reading post injection, which consisted of the raised bleb on the skin from product administration. A reactogenic classification of a score of grade 3 or greater was recorded as an adverse event.

In the Phase I safety non-endemic study, of the ten participants tested with titrated doses of MLSA-LAM or MLCwA, only one subject elicited a DTH skin test response of induration against the 2.5 µg dose of MLCwA at 72 h. Induration measurements at 48 and 72 h were very similar and since the 48 h reading was dropped from the Phase II study, only the 72 h values are reported. Reactogenicity results indicated that the test antigens were well tolerated at all doses, but the 2.5 µg dose of both MLSA-LAM and MLCwA was responsible for 8 of 15 of erythema events and one of each induration or itching events noted. Although these were expected reactions for skin testing, they were not expected in healthy controls; therefore, as a precaution, the 2.5 µg dose was dropped from further testing. The final recommendations from the safety non-endemic study were to test the new leprosy skin test antigens in an endemic region for leprosy at 1.0 µg and 0.1 µg doses only.

In the Phase II safety trial in Nepal involving Stages A (10 subjects) and B (90 subjects) trial, individual subjects who were randomly assigned to a skin test antigen group, were reassigned a sequential number for reporting data (**Table S1**). Of participants tested in the Stage A study, only one subject in each group elicited a DTH skin test response of induration against the high (1.0 µg) dose of MLSA-LAM and MLCwA. Three subjects exhibited erythema against both antigens at the high dose, and one subject exhibited erythema against the low dose of MLCwA only. Itching was observed in two subjects with the high dose of each antigen (reactogenicity data on individual subjects is available from the lead authors).

Of the ninety participants tested in the Phase II, Stage B safety study, twelve subjects each elicited induration, and ten and eleven subjects showed erythema for the high dose of MLSA-LAM and MLCwA, respectively. Only one and two subjects showed erythema and induration at the low dose of both antigens, respectively. Itching was observed only in one subject at the high dose of MLCwA, and pain was observed in three or fewer subjects each at the high and low dose of MLSA-LAM and MLCwA.

Stage C-1a (high dose) and C-1b (low dose) 72 h induration measurements are provided as a supplement in **Table S2**, since this aspect of the study involved both safety and efficacy in the leprosy patients, contacts, and TB patients and most reactions consisted of mild to moderate induration and erythema, with only a few cases of mild pruritis and pain and one case of urticaria, infection, and blistering with MLSA-LAM at both the high and low dose. One case of bleeding at the site of injection was seen with MLSA-LAM at the low dose. The HC and TB groups had the highest number of reactions in the high dose study, whereas the BT/TT and HC groups had more reactions in the low dose study. The BL/LL group had the lowest number of reactions across both the high and low dose of each leprosy antigens.

#### Adverse events

For the Phase II clinical trial, each adverse event (AE) was graded and coded by the MedDRA SOC [14]. Only one mild adverse event recorded as lymphangitis was listed as possibly being related to one of the study products. The subject who experienced this AE was administered MLCwA and PPD. Five AE, including two serious adverse events (SAE), were deemed unrelated to the study antigens as determined by the Clinical Investigator and Medical Monitor. One mild AE described as a type I hypersensitivity reaction was observed in Stage C-1b in a BT/TT subject on day 12. The subject was given prednisolone, and the event was ongoing upon study termination. All other AEs were related to PPD reactions greater than 30 mm. Of the two SAEs, one subject, a 28 year old male with no past medical history, was hospitalized for appendicitis after the 28 day study period. This individual underwent an appendectomy on day 34 for appendicitis. The second SAE involved a death due to cerebral hemorrhage, possibly secondary to an A-V (arterio-venous) aneurysm. The subject was a 21 year old male with unknown personal or family medical history. The subject was on concomitant medications. The subject's condition deteriorated and he died on day 25 of the study. Both the investigator and medical monitor assessed these events and deemed them unrelated to the study antigen.

### Efficacy

#### Baseline

**Figure 2:**
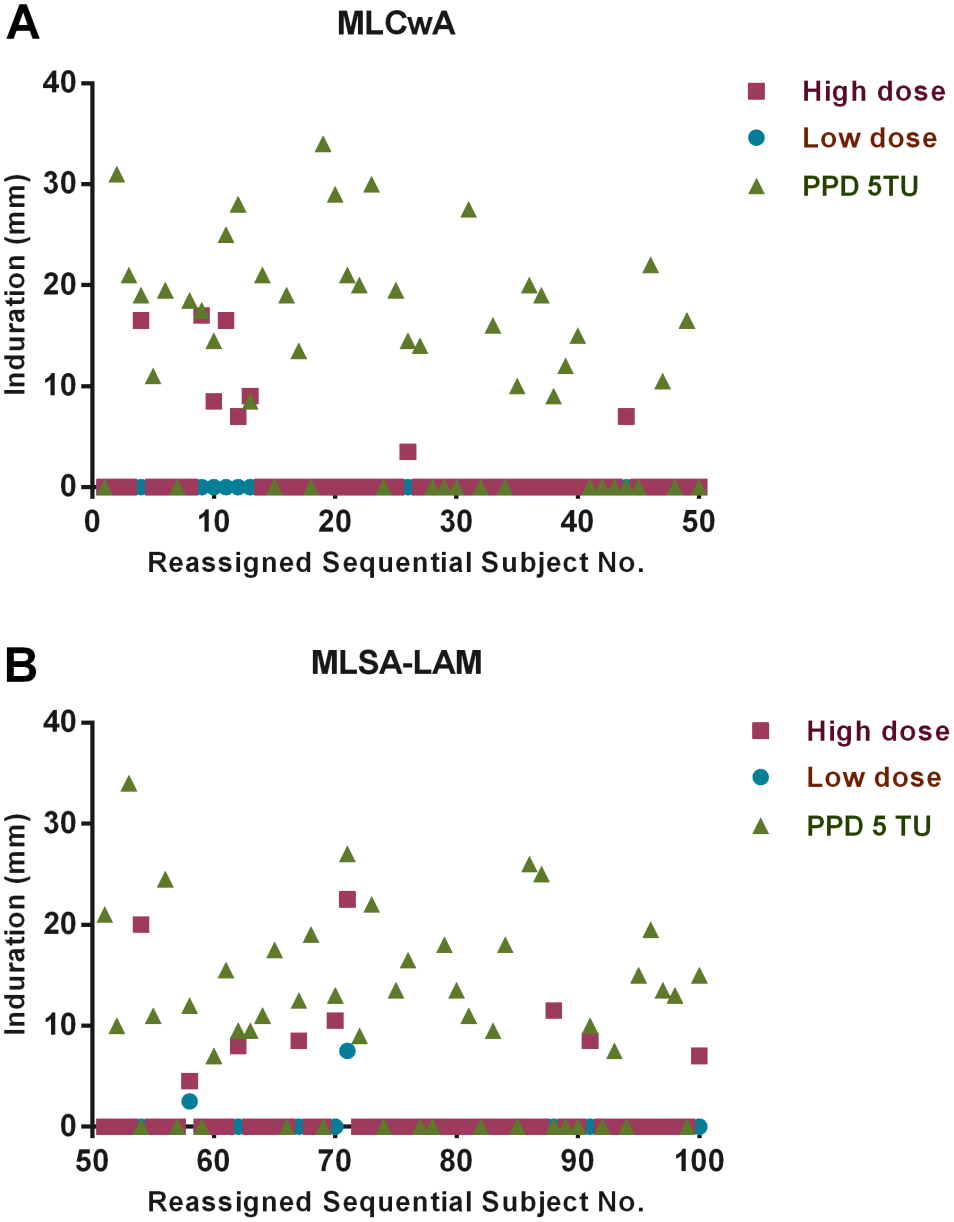
Phase II, Stage A/B – DTH induration by subject. Phase II, Stage A/B graph depicting DTH indurations elicited by leprosy skin test antigens at the high dose (1.0 µg) and low dose (0.1 µg), and PPD at 5 TU: A) MLCwA, and B) MLSA-LAM. The first five subjects on both graphs represent subjects from Stage A, and the remaining 45 subjects on both graphs represent subjects from Stage B.

A baseline was derived for each antigen at each dose based on Phase II, Stage A/B data from healthy controls in an endemic region without known exposure to leprosy. **Figure 2** shows the response to MLCwA high and low doses from the first five subjects in Stage A and the first 45 subjects in Stage B and MLSA-LAM high and low doses from the second five subjects from Stage A and the second 45 subjects from Stage B, each compared to PPD. Three individuals responded to one or the other leprosy skin test antigens only, and 14 responded to a leprosy antigen and PPD; whereas, 53 volunteers responded to PPD only.

#### Relationship to BCG vaccination

Since tuberculosis is prevalent in Nepal and many individuals were vaccinated with BCG, most subjects were expected to respond to PPD; hence, PPD testing was a measure of not only exposure to tuberculosis, but also BCG vaccination, and, to some extent, non-pathogenic environmental mycobacteria. In all stages of the Phase II trial, BCG vaccination scars were found in 163/260 (63%) subjects: 77/100 (77%) endemic controls (EC); 28/40 (70%) HC; 27/40 (68%) TB; 12/40 (30%) BT/TT; and, 19/40 (48%) BL/LL. The mean size of the largest scar across all subjects was 10±4 mm, with a range of 2–33 mm. The mean number of scars was 1.0±0.3 with a range of 1–3. BCG vaccination history was compared to skin test responses in EC. Of vaccinated subjects, 15 of 77 (19%) responded to the skin test antigens; whereas 61 of 77 (79%) responded to PPD. In contrast, of non-vaccinated subjects, 2 of 23 (7%) responded to the skin test antigens and 13 of 23 (57%) elicited a response to PPD.

#### Induration size relative to PPD

Of the EC subjects who reacted to both the test antigen (high and low dose) and PPD, a correlation between the size of the DTH reaction was observed when comparing MLSA-LAM to PPD (r^2^ = 0.90), but not when comparing MLCwA to PPD (r^2^ = 0.25). The frequency of subjects responding to leprosy skin test antigens normalized to PPD is shown in **Table 1**. The majority of subjects (63%) did not respond to either skin test antigen; however, of those individuals who did respond, the most frequent induration size relative to PPD was less than 10 mm. Individuals with a higher PPD response did not necessarily evoke a response from the leprosy antigens.

**Table 1:** Phase II, Stage A/B: Frequency of induration size (EC relative to PPD).

	MLCwA		MLSA-LAM		No. *M. lep* Responders^b^	No. PPD Responders	Ratio *M.lep*/PPD	Frequency (%)
Induration Range (mm)	High Dose	Low Dose	High Dose	Low Dose				
0.0	42	50	41	48	83	33	83/33 (2.52)	2.52/4.03 (63)
0.1 to 9.9	5	0	5	2^a^	10	8	10/8 (1.25)	1.25/4.03 (31)
10.0 to 19.9	3	0	2	0	5	39	5/39 (0.13)	0.13/4.03 (3)
20.0 to 29.9	0	0	2	0	2	16	2/16 (0.13)	0.13/4.03 (3)
>30.0	0	0	0	0	0	4	0/4 (0.00)	0.00/4.03 (0)
Total	50		50		100	100	(4.03)	100

a Subjects responding to MLSA-LAM low dose also responded to MLSA-LAM high dose.

b The number of responders against *M. leprae* skin test antigens is derived from the high dose of both antigens.

#### Definition of a positive response

**Figure 3:**
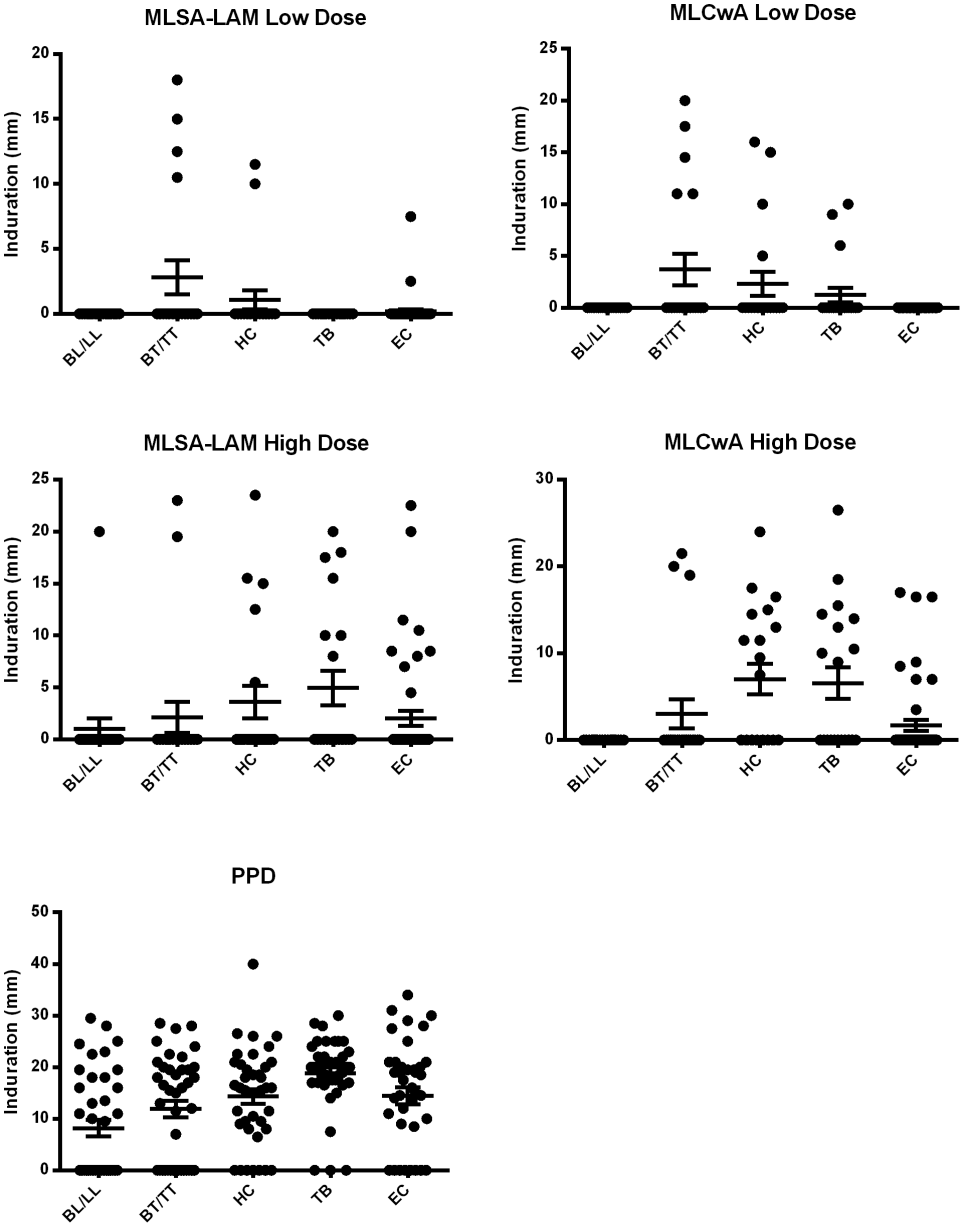
Dot plot of induration measurements. Induration results are provided across five subject groups, including BL/LL leprosy patients (*n* = 19 in low and *n* = 20 in high dose group), BT/TT leprosy patients (*n* = 20), HC (*n* = 20), TB (*n* = 20), and ECs (*n* = 50). Low and high dose groups were combined to show PPD responses: BL/LL leprosy patients (*n* = 39) and all other groups (*n* = 40). Mean and standard deviation are shown.

Induration measurements from Stage C-1a (high dose) and C-1b (low dose) have been graphed on a dot plot across cohorts tested with MLSA-LAM and MLCwA (**Figure 3**). There was a near total lack of response of the BL/LL subjects to the skin test antigens, yet a vigorous response to PPD. The leprosy antigens were behaving according to precedent in that respect [33].

**Figure 4:**
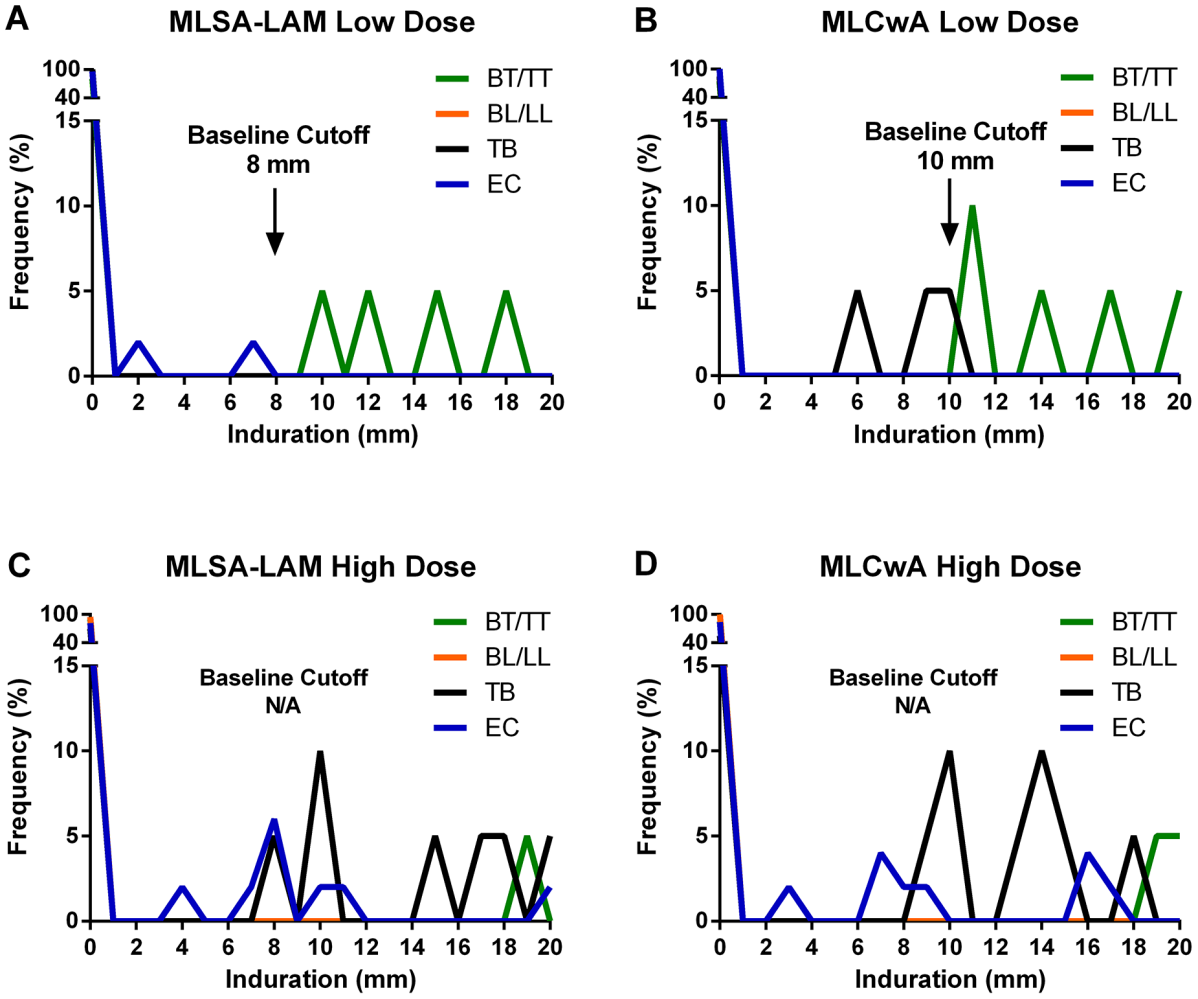
Distribution frequency of induration. Frequency distribution graphs were used to establish cut off points for each skin test antigen at each dosage tested: A) MLSA-LAM low dose, B) MLCwA low dose, C) MLSA-LAM high dose, D) MLCwA high dose. Frequency of induration reaction (mm) of EC and TB groups were graphed against BT/TT and BL/LL leprosy groups. The anti-mode between the control and leprosy patient group represents the cut off for each antigen and antigen dose.

A frequency distribution of induration size was used to compare BT/TT to EC and TB groups to identify a cutoff point. The distribution curve shown in **Figure 4** was difficult to interpret due to limited sample size and few reactors in the BT/TT groups. The EC response served as the baseline, while the TB response provided the worst case scenario with individuals infected with a related mycobacterial species. The projected cut off point is at the anti-mode, or the point at which the control groups no longer respond and the patient groups begin responding. MLSA-LAM and MLCwA low dose presented an anti-mode at 8 mm and 10 mm, respectively. The curves for the high dose antigens did not present a biomodal distribution; therefore, a cutoff point could not be determined. ROC curve analysis calculated the cut off point for MLSA-LAM and MLCwA low dose to be greater than 5.2 mm and 9.5 mm, respectively. The likelihood ratios were high; however, p-values were not significant (p = 0.28 and 0.46, respectively) due to limited BT/TT group responses. A larger sample size is needed to properly evaluate this parameter.

#### Proportion of positive reactors

The proportion of positive reactors and mean induration for each study group were directly compared to the EC group. Results are shown as supporting information in **Table 2**. The low dose of MLSA-LAM elicited the strongest response in BT/TT subjects (4/20) 20% and HC subjects (2/20) 10% compared to EC subjects (2/50) 4% and TB subjects (0/20) 0%. The high doses of both leprosy skin test antigens elicited a greater response in HC and TB patients than BT/TT leprosy patients. One BL/LL subject in the MLSA-LAM high dose group reacted with an induration of 20 mm. This subject was a 46 year old male with LL leprosy, who had been treated with MDT for a period of one month before enrolling in the high dose study. This participant was smear positive, had a bacterial index of 4.0, did not have a history of Type I or ENL reactions, had a single BCG scar, and, was using a topical steroid ointment for the treatment of mild eczema on two fingers. Comparison of mean induration measurements across all subjects showed a higher response in the BT/TT leprosy patients compared to HC with both antigens at the low dose. Antigens at the high dose showed a lower response in BT/TT leprosy patients compared to HC and TB patients. A significant increase in the number of responders and mean induration in TB patients and HC was seen with PPD, with fewer BL/LL leprosy patients responding overall.

**Table 2:** Number of positive responders and mean induration compared to ECs.

Antigen	Dose	Group	Number of Subjects		Mean Induration	
			No. Pos/Total (%)	Ratio (Test/EC)	Mean (mm)	Ratio (Test/EC)
MLSA-LAM	Low	EC	2/50 (4)	na	0.2	na
		BT/TT	4/20 (20)	20/4 (5.0)	2.8	2.8/0.2 (14.0)
		BL/LL	0/19(0)	0/4 (0.0)	0.0	0.0/0.2 (0.0)
		HC	2/20 (10)	10/4 (2.5)	1.1	1.1/0.2 (5.5)
		TB	0/20 (0)	0/4 (0.0)	0.0	0.0/0.2 (0.0)
MLCwA	Low	EC	0/50 (1^a^)	na	0.1^a^	na
		BT/TT	5/20 (25)	25/1 (25.0)	3.7	3.7/0.1 (37.0)
		BL/LL	0/19 (0)	0/1 (0.0)	0.0	0.0/0.1 (0.0)
		HC	4/20 (20)	20/1 (20.0)	2.3	2.3/0.1 (23.0)
		TB	3/20 (15)	15/1 (15.0)	1.3	1.3/0.1 (13.0)
MLSA-LAM	High	EC	10/50 (20)	na	2.0	na
		BT/TT	2/20 (10)	10/20 (0.5)	2.1	2.1/2.0 (1.1)
		BL/LL	1/20 (5)	5/20 (0.3)	1.0	1.0/2.0 (0.5)
		HC	5/20 (25)	25/20 (1.3)	3.6	3.6/2.0 (1.8)
		TB	7/20 (35)	35/20 (1.8)	5.0	5.0/2.0 (2.5)
MLCwA	High	EC	8/50 (16)	na	1.7	na
		BT/TT	3/20 (15)	15/16 (0.9)	3.0	3.0/1.7 (1.8)
		BL/LL	0/20 (0)	0/16 (0.0)	0.0	0.0/1.7 (0.0)
		HC	10/20 (50)	50/16 (3.1)	7.0	7.0/1.7 (4.1)
		TB	9/20 (45)	45/16 (2.8)	6.6	6.6/1.7 (3.9)
PPD	5 TU	EC	67/100 (67)	na	11.4	na
	2 TU	BT/TT	25/40 (63)	63/67 (94)	11.9	11.9/11.4 (1.0)
	2 TU	BL/LL	18/39 (46)	46/67 (69)	8.2	8.2/11.4 (0.7)
	2 TU	HC	33/40 (83)	83/67 (124)	14.3	14.3/11.4 (1.3)
	2 TU	TB	37/40 (93)	93/67 (139)	18.9	18.9/11.4 (1.7)

a To allow calculations, the EC percent positive has been changed to 1.0.

#### Antigen correlation

With different responses being recognized between the two skin test antigens and dosages, results were compared by linear regression to look for correlations using the BT/TT group, albeit with few responders. The highest correlation was found between the two leprosy antigens at the low dose with a covariance (r^2^) value of 0.81, followed by the high dose with a covariance of 0.67. There was no correlation between MLSA-LAM high and low dose, or either antigen at either dose against PPD.

#### Sensitivity and specificity

Diagnostic statistics provide a measurable assessment of the leprosy skin test antigens [34]. Four statistics provide the foundation for assessing a diagnostic test: 1) sensitivity; 2) specificity; 3) positive predictive value; and, 4) negative predictive value. Generally, a good diagnostic test is both sensitive and specific [35]. Sensitivity and specificity statistics have been calculated for the two new antigens and antigen doses compared to PPD in **Table 3**. Caution was taken when interpreting these values, because of the small sample sizes and limited BT/TT responders; PPV and NPV were not calculated due to limited positive responders. Results showed that MLSA-LAM and MLCwA at the low dose were highly specific (100% and 95%), but lacked sensitivity (20% and 25%). PPD as a diagnostic for tuberculosis was sensitive (90%), but not specific (41%). Sensitivity of skin test antigens must be enhanced to develop a viable diagnostic test for leprosy. Preliminary results from *in-vitro* whole blood IFNγ release assays (WB-IGRA) shown in **Table 4**, showed slightly improved sensitivity while retaining specificity at the high dose of each antigen; though full analysis of this data is not yet completed. A comprehensive comparison of the adaptive T-cell response against these two leprosy antigens by skin test and WB-IGRA, and serological response against PGL-I will be published soon.

**Table 3:** Diagnostic test statistics – Skin test.

Antigen	Dose	Sensitivity		Specificity	
		(BT/TT)	(BL/LL)	(EC)	(TB)
MLSA-LAM	Low	(4/20) 20%	(0/19) 0%	(50/50) 100%	(20/20) 100%
MLCwA	Low	(5/20) 25%	(0/19) 0%	(50/50) 100%	(19/20) 95%
MLSA-LAM	High	(2/20) 10%	(1/20) 5%	(43/50) 86%	(14/20) 70%
MLCwA	High	(3/20) 15%	(0/20) 0%	(47/50) 94%	(12/20) 60%

Diagnostic test statistics were calculated for each test method. Sensitivity (Se) is the likelihood to detect the presence of disease [Total Positive (TP)/TP + False Negative (FN)]. Specificity (Sp) is the likelihood to detect absence of disease [(Total Negative (TN)/TN + False Positive (FP)]. PPD served as an antigen control. Statistics for detecting tuberculosis: Sensitivity is (36/40) 90%, specificity is (41/100) 41%.

**Table 4:** Diagnostic test statistics – WB-IGRA^a^.

Antigen	Dose	Cut-off (IU/ml)	Sensitivity (BT/TT)	Specificity (TB)	P value
MLSA-LAM	Low	1.30	83%	53%	0.150
MLCwA	Low	1.20	72%	53%	0.500
MLSA-LAM	High	0.14	47%	95%	0.001
MLCwA	High	0.22	32%	95%	0.030

a Preliminary unpublished results.

## Discussion

Study results were presented as trends, due to small scale sample sizes. Both antigens and antigen dosages are safe for use in BT/TT and BL/LL leprosy patients, HC of lepromatous leprosy patients, and TB patients. The diagnostic accuracy of both skin test antigens at the low dose (0.1 µg) was found to be inadequate in terms of sensitivity, but acceptable in terms of specificity. MLSA-LAM was shown to have slightly higher specificity than MLCwA at the low dose when comparing BT/TT leprosy patients against individuals infected with *M. tuberculosis*. At the high dose (1.0 µg), both antigens were limited in both sensitivity and specificity. Leprosy skin test antigens were found to be unresponsive in BL/LL leprosy patients confirming *M. leprae* specific anergy, yet capable of eliciting a response in some HC of BL/LL leprosy patients. A cut-off point for each antigen and antigen dose was calculated, but not with significance, due to limited positive responders in the BT/TT leprosy group.

Sensitivity and specificity were lacking with the Rees and Convit soluble antigens when tested in 2,602 Indian subjects [29]. A biomodal distribution of induration was seen with both antigens, but newly diagnosed leprosy patients, contacts, and non-contacts responded equally. In Northern Malawi, 15,630 subjects were tested with 5 batches of the Rees MLSA antigen prepared from two protocols [36]. With first and second generation antigens, a response from paucibacillary (similar to BT/TT) [37] leprosy patients was seen in 76% and 38% of the subjects, respectively; however, ECs responded in 42% and 32% of the subjects, resulting in a difference of 34% and 6% detection rates, respectively. These percentages represent responders over baseline and are close to the 10–20% detection rates seen with low dose MLSA-LAM and MLCwA antigens.

The low detection rate of known BT/TT leprosy patients with these antigens suggests that they are not suitable for detection of clinical leprosy. They do, however, elicit a response in 10–20% of HC, suggesting that they might be suitable for detection of pre-symptomatic leprosy [38]. The proportion of positive HC responding in these studies was consistent with documented risk of infection from a high bacillary index case at one in seven (14%) of 178 households studied [39]. Previous WB-IGRA studies with MLSA-LAM and MLCwA showed nearly identical results to these skin test studies, except that HC responded with a higher geometric mean than BT/TT leprosy patients; EC and TB patients did not respond [40]. These authors also found that recent exposure resulted in substantially stronger responses. Detection of BT/TT leprosy patients who were under treatment or completed treatment (51%) may have affected sensitivity results in these studies.

At the high dose compared to the low dose, both antigens elicited a response in a higher number of HC, TB, and EC subjects, but the number of BT/TT responders remained the same. These phenomena may be related to that observed when Leprosin A (Rees antigen) was shown to immunologically suppress the skin test response to PPD in both BT/TT and BL/LL leprosy patients [41]. These data support the idea that there may be a difference in the antigenic profile that stimulates a response in pre-symptomatic, but not symptomatic leprosy.

The immunological environment of early leprosy is unknown; however, advances have been made in understanding the innate and adaptive immune mediated pathways that promote and control disease pathology [42], [43]. In tuberculosis, the infection delays onset of adaptive immunity, which provides a window to establish a successful infection. Disease progression in tuberculosis, like leprosy, is then dependent on the immunological status of the host [44]. Striking similarities of the immunology and pathology between these two diseases suggest that TT leprosy could be a latent form of disease, under the control of the immune system, whereas LL leprosy is known to be the active form of disease with T-cell hyporesponsiveness [45] Borderline forms are immunologically unstable and can downgrade depending on the immunological position of the host [33]. This continuum of immunological events probably occurs prior to and during manifestation of clinical symptoms, providing opportunities for an early clinical diagnostic tool.

Antigen specificity at the low dose was thought to be related to the removal of lipoglycans, including the immunosuppressive and cross-reactive LAM, lipomannan (LM), and phosphatidylinositol mannoside (PIM), and other lipids and lipoproteins [46]–[48]. Remaining proteins were numerous, but many shared sequence homology with *M. tuberculosis* [49]. Nonetheless, of the 100 EC tested, 77% had been vaccinated with BCG and 67% reacted with PPD, while only 2% reacted to the low dose leprosy antigens (2 with MLSA-LAM and 0 with MLCwA). This suggests that the leprosy antigens are detecting specific CMI responses resulting from an infection with *M. leprae*, implying that transmission has occurred in these few healthy subjects. Of the 20 TB subjects tested in the Phase II, Stage C-1b study, 95% (n = 19) reacted to PPD, but only 10% (n = 2) reacted to MLCwA and none reacted to MLSA-LAM. Another possibility is that the dose alone, or in combination with the removal of lipoglycans, resulted in high specificity. At the low dose, *M. leprae* specific proteins may be available for recognition, whereas at the high dose, those same proteins may be overpowered by ubiquitous mycobacterial proteins lending to cross-reactive responses with TB patients and EC exposed to environmental mycobacteria or vaccinated with BCG [20].

The strength of these studies was in the verification that new refined leprosy skin test antigens were immunologically active in BT/TT leprosy patients, anergic in BL/LL leprosy patients, and highly specific in BT/TT leprosy patients. The skin test method was simple, easy for field use, and minimally invasive, potentially affording a feasible early diagnostic test tool. Limitations of these studies were difficulties in shipping materials through customs, lengthy document review and approvals, multiple stages in the Phase II protocol; prolonged duration to complete the study; political turmoil in the endemic country; intermittent communication services; and above all, unacceptably low sensitivity to warrant the larger scale trial originally planned.

Our findings do not support further work on the skin test method with MLSA-LAM and MLCwA. The future of diagnostic tests for pre-symptomatic leprosy needs to be specific, but foremost, must be sensitive to detect early infection with *M. leprae*. Focus on the early T-cell response using multiple *M. leprae* specific antigens and immunological biomarkers may be required to enhance sensitivity. Promising proteins and peptides using cellular biomarkers to detect leprosy [38], [50] and tuberculosis [51] have been recently reported. Comparable dose-dependent *in-vitro* studies with MLSA-LAM and MLCwA, as native antigens, would be expected to offer specificity and may improve sensitivity. Adding serology tests with specific antigens such as phenolic glycolipid-I (PGL-I) may increase early detection rates for BL/LL leprosy patients [52], [53]. Despite the choice of test method, identification of the target population for pre-symptomatic leprosy, i.e. HC as identified in the COLEP study [54]–[56], will need to be determined. Testing in a large scale randomized study with follow-up will undoubtedly be required to reveal whether a future diagnostic test could tip the balance toward interrupting transmission.

## Supporting Information

Checklist S1 **CONSORT checklist.**
(DOC)

Checklist S2 **STARD checklist.**
(DOC)

Table S1 **Phase II, Stage A/B - Induration measurements at 72 h.** One hundred and one volunteers were recruited for Stage A and B, because one declined participation. A) In the Phase II, Stage A study, the 48 and 72 h induration measurements were very similar, and since the 48 hour response was dropped from Stage B, only the 72 hour values are provided for comparison. B) Phase II, Stage B study 72 h induration measurements. Response to skin test antigens: (*) a total of 30 individuals did not respond to either the intervention or to PPD, (**) a total of 3 individuals responded to one or the other antigens, but not PPD, (***) a total of 53 individuals responded to Tuberculin PPD only, and (****) a total of 14 individuals responded to both the intervention and PPD.
(DOCX)

Table S2 **Phase II, Stage C-1a/b (antigen high/low dose) induration at 72 h.** Response patterns for individual subjects have been marked (*) no reaction to either intervention or PPD, (**) reaction to one or both interventions, but not to PPD, (***) reaction to PPD only, and (****) reaction to one or both intervention and PPD.
(DOCX)
