# Correction: Safety and Efficacy Assessment of Two New Leprosy Skin Test Antigens: Randomized Double Blind Clinical Study

**DOI:** 10.1371/journal.pntd.0003065

**Published:** 2014-07-07

**Authors:** The PLOS Neglected Tropical Diseases Staff

The eighth author's name is spelled incorrectly. The correct name is: Chhatra B. Kunwar.
