# Supplementary material for: Safety and Efficacy Assessment of Two New Leprosy Skin Test Antigens: Randomized Double Blind Clinical Study
Source: PLoS Negl Trop Dis. 2014 May 29;8(5):e2811. doi: 10.1371/journal.pntd.0002811 (PMC4038488; doi:10.1371/journal.pntd.0002811)
Supplement: Table S1 — Phase II, Stage A/B - Induration measurements at 72 h. One hundred and one volunteers were recruited for Stage A and B, because one declined participation. A) In the Phase II, Stage A study, the 48 and 72 h induration measurements were very similar, and since the 48 hour response was dropped from Stage B, only the 72 hour values are provided for comparison. B) Phase II, Stage B study 72 h induration measurements. Response to skin test antigens: (*) a total of 30 individuals did not respond to either the intervention or to PPD, (**) a total of 3 individuals responded to one or the other antigens, but not PPD, (***) a total of 53 individuals responded to Tuberculin PPD only, and (****) a total of 14 individuals responded to both the intervention and PPD. (DOCX) [file pntd.0002811.s003.docx]

## Supporting Information

**Table S1. Phase II, Stage A/B - Induration Measurements at 72 h.**

A) Phase II, Stage A

| Assigned Sequential Subject No. | Actual Subject ID | Intervention | Induration (mm) | | | |  |
| --- | --- | --- | --- | --- | --- | --- | --- |
|  |  |  | Intervention | | Tuberculin | Saline |  |
|  |  |  | 0.1 µg | 1.0 µg | 5TU | N/A |  |
| 1 | 2 | MLCwA | 0 | 0 | 0 | 0 | * |
| 2 | 3 | MLCwA | 0 | 0 | 31 | 0 | *** |
| 3 | 5 | MLCwA | 0 | 0 | 21 | 0 | *** |
| 4 | 6 | MLCwA | 0 | 16.5 | 19 | 0 | **** |
| 5 | 9 | MLCwA | 0 | 0 | 11 | 0 | *** |
| 51 | 1 | MLSA-LAM | 0 | 0 | 21 | 0 | *** |
| 52 | 4 | MLSA-LAM | 0 | 0 | 10 | 0 | *** |
| 53 | 7 | MLSA-LAM | 0 | 0 | 34 | 0 | *** |
| 54 | 8 | MLSA-LAM | 0 | 20 | 0 | 0 | ** |
| 55 | 10 | MLSA-LAM | 0 | 0 | 11 | 0 | *** |

B) Phase II, Stage B

| Assigned Sequential Subject No. | Actual Subject ID | Intervention | Induration (mm) | | |  |
| --- | --- | --- | --- | --- | --- | --- |
|  |  |  | Intervention | | Tuberculin |  |
|  |  |  | 0.1 µg | 1.0 µg | 5TU |  |
| 6 | 12 | MLCwA | 0 | 0 | 19.5 | *** |
| 7 | 16 | MLCwA | 0 | 0 | 0 | * |
| 8 | 18 | MLCwA | 0 | 0 | 18.5 | *** |
| 9 | 19 | MLCwA | 0 | 17 | 17.5 | **** |
| 10 | 20 | MLCwA | 0 | 8.5 | 14.5 | **** |
| 11 | 22 | MLCwA | 0 | 16.5 | 25 | **** |
| 12 | 24 | MLCwA | 0 | 7 | 28 | **** |
| 13 | 25 | MLCwA | 0 | 9 | 8.5 | **** |
| 14 | 26 | MLCwA | 0 | 0 | 21 | *** |
| 15 | 29 | MLCwA | 0 | 0 | 0 | * |
| 16 | 33 | MLCwA | 0 | 0 | 19 | *** |
| 17 | 34 | MLCwA | 0 | 0 | 13.5 | *** |
| 18 | 35 | MLCwA | 0 | 0 | 0 | * |
| 19 | 36 | MLCwA | 0 | 0 | 34 | *** |
| 20 | 40 | MLCwA | 0 | 0 | 29 | *** |
| 21 | 41 | MLCwA | 0 | 0 | 21 | *** |
| 22 | 42 | MLCwA | 0 | 0 | 20 | *** |
| 23 | 44 | MLCwA | 0 | 0 | 30 | *** |
| 24 | 47 | MLCwA | 0 | 0 | 0 | * |
| 25 | 50 | MLCwA | 0 | 0 | 19.5 | *** |
| 26 | 53 | MLCwA | 0 | 3.5 | 14.5 | **** |
| 27 | 54 | MLCwA | 0 | 0 | 14 | *** |
| 28 | 56 | MLCwA | 0 | 0 | 0 | * |
| 29 | 57 | MLCwA | 0 | 0 | 0 | * |
| 30 | 59 | MLCwA | 0 | 0 | 0 | * |
| 31 | 64 | MLCwA | 0 | 0 | 27.5 | *** |
| 32 | 65 | MLCwA | 0 | 0 | 0 | * |
| 33 | 66 | MLCwA | 0 | 0 | 16 | *** |
| 34 | 68 | MLCwA | 0 | 0 | 0 | * |
| 35 | 70 | MLCwA | 0 | 0 | 10 | *** |
| 36 | 71 | MLCwA | 0 | 0 | 20 | *** |
| 37 | 72 | MLCwA | 0 | 0 | 19 | *** |
| 38 | 75 | MLCwA | 0 | 0 | 9 | *** |
| 39 | 79 | MLCwA | 0 | 0 | 12 | *** |
| 40 | 80 | MLCwA | 0 | 0 | 15 | *** |
| 41 | 81 | MLCwA | 0 | 0 | 0 | * |
| 42 | 82 | MLCwA | 0 | 0 | 0 | * |
| 43 | 85 | MLCwA | 0 | 0 | 0 | * |
| 44 | 86 | MLCwA | 0 | 7 | 0 | ** |
| 45 | 90 | MLCwA | 0 | 0 | 0 | * |
| 46 | 91 | MLCwA | 0 | 0 | 22 | *** |
| 47 | 94 | MLCwA | 0 | 0 | 10.5 | *** |
| 48 | 97 | MLCwA | 0 | 0 | 0 | * |
| 49 | 99 | MLCwA | 0 | 0 | 16.5 | *** |
| 50 | 100 | MLCwA | 0 | 0 | 0 | * |
| 56 | 11 | MLSA-LAM | 0 | 0 | 24.5 | *** |
| 57 | 13 | MLSA-LAM | 0 | 0 | 0 | * |
| 58 | 14 | MLSA-LAM | 2.5 | 4.5 | 12 | **** |
| 59 | 15 | MLSA-LAM | 0 | 0 | 0 | * |
| 60 | 17 | MLSA-LAM | 0 | 0 | 7 | *** |
| 61 | 21 | MLSA-LAM | 0 | 0 | 15.5 | *** |
| 62 | 23 | MLSA-LAM | 0 | 8 | 9.5 | **** |
| 63 | 27 | MLSA-LAM | 0 | 0 | 9.5 | *** |
| 64 | 28 | MLSA-LAM | 0 | 0 | 11 | *** |
| 65 | 30 | MLSA-LAM | 0 | 0 | 17.5 | *** |
| 66 | 31 | MLSA-LAM | 0 | 0 | 0 | * |
| 67 | 32 | MLSA-LAM | 0 | 8.5 | 12.5 | **** |
| 68 | 37 | MLSA-LAM | 0 | 0 | 19 | *** |
| 69 | 38 | MLSA-LAM | 0 | 0 | 0 | * |
| 70 | 39 | MLSA-LAM | 0 | 10.5 | 13 | **** |
| 71 | 43 | MLSA-LAM | 7.5 | 22.5 | 27 | **** |
| 72 | 45 | MLSA-LAM | 0 | 0 | 9 | *** |
| 73 | 46 | MLSA-LAM | 0 | 0 | 22 | *** |
| 74 | 48 | MLSA-LAM | 0 | 0 | 0 | * |
| 75 | 49 | MLSA-LAM | 0 | 0 | 13.5 | *** |
| 76 | 51 | MLSA-LAM | 0 | 0 | 16.5 | *** |
| 77 | 52 | MLSA-LAM | 0 | 0 | 0 | * |
| 78 | 55 | MLSA-LAM | 0 | 0 | 0 | * |
| 79 | 58 | MLSA-LAM | 0 | 0 | 18 | *** |
| 80 | 60 | MLSA-LAM | 0 | 0 | 13.5 | *** |
| 81 | 61 | MLSA-LAM | 0 | 0 | 11 | *** |
| 82 | 62 | MLSA-LAM | 0 | 0 | 0 | * |
| 83 | 63 | MLSA-LAM | 0 | 0 | 9.5 | *** |
| 84 | 67 | MLSA-LAM | 0 | 0 | 18 | *** |
| 85 | 69 | MLSA-LAM | 0 | 0 | 0 | * |
| 86 | 73 | MLSA-LAM | 0 | 0 | 26 | *** |
| 87 | 74 | MLSA-LAM | 0 | 0 | 25 | *** |
| 88 | 76 | MLSA-LAM | 0 | 11.5 | 0 | ** |
| 89 | 77 | MLSA-LAM | 0 | 0 | 0 | * |
| 90 | 78 | MLSA-LAM | 0 | 0 | 0 | * |
| 91 | 83 | MLSA-LAM | 0 | 8.5 | 10 | **** |
| 92 | 84 | MLSA-LAM | 0 | 0 | 0 | * |
| 93 | 87 | MLSA-LAM | 0 | 0 | 7.5 | *** |
| 94 | 89 | MLSA-LAM | 0 | 0 | 0 | * |
| 95 | 92 | MLSA-LAM | 0 | 0 | 15 | *** |
| 96 | 93 | MLSA-LAM | 0 | 0 | 19.5 | *** |
| 97 | 95 | MLSA-LAM | 0 | 0 | 13.5 | *** |
| 98 | 96 | MLSA-LAM | 0 | 0 | 13 | *** |
| 99 | 98 | MLSA-LAM | 0 | 0 | 0 | * |
| 100 | 101 | MLSA-LAM | 0 | 7 | 15 | **** |
