# Supplementary material for: Safety and Efficacy Assessment of Two New Leprosy Skin Test Antigens: Randomized Double Blind Clinical Study
Source: PLoS Negl Trop Dis. 2014 May 29;8(5):e2811. doi: 10.1371/journal.pntd.0002811 (PMC4038488; doi:10.1371/journal.pntd.0002811)
Supplement: Table S2 — Phase II, Stage C-1a/b (antigen high/low dose) induration at 72 h. Response patterns for individual subjects have been marked (*) no reaction to either intervention or PPD, (**) reaction to one or both interventions, but not to PPD, (***) reaction to PPD only, and (****) reaction to one or both intervention and PPD. (DOCX) [file pntd.0002811.s004.docx]

## Supporting Information

## Table S2. Phase II, Stage C-1a/b (Antigen High/Low Dose) Induration at 72 h.

1. Phase II, Stage C-1a (High Dose)

| Stage C-1 a | BT/TT | | | | Stage C-1 a | BL/LL | | | |
| --- | --- | --- | --- | --- | --- | --- | --- | --- | --- |
| Subject No. | MLCwA | MLSA-LAM | PPD | Reaction | Subject No. | MLCwA | MLSA-LAM | PPD | Reaction |
| 1 | 0 | 0 | 18 | *** | 1 | 0 | 20 | 0 | ** |
| 2 | 0 | 0 | 19.5 | *** | 2 | 0 | 0 | 0 | * |
| 3 | 0 | 0 | 0 | * | 3 | 0 | 0 | 29.5 | *** |
| 4 | 0 | 0 | 28.5 | *** | 4 | 0 | 0 | 28 | *** |
| 5 | 0 | 0 | 0 | * | 5 | 0 | 0 | 0 | * |
| 6 | 21.5 | 23 | 27.5 | **** | 6 | 0 | 0 | 11 | *** |
| 7 | 0 | 0 | 11.5 | *** | 7 | 0 | 0 | 0 | * |
| 8 | 0 | 0 | 28 | *** | 8 | 0 | 0 | 18 | *** |
| 9 | 0 | 0 | 0 | * | 9 | 0 | 0 | 19.5 | *** |
| 10 | 0 | 0 | 24 | *** | 10 | 0 | 0 | 16 | *** |
| 11 | 0 | 0 | 18.5 | *** | 11 | 0 | 0 | 11 | *** |
| 12 | 0 | 0 | 19.5 | *** | 12 | 0 | 0 | 22.5 | *** |
| 13 | 0 | 0 | 19.5 | *** | 13 | 0 | 0 | 0 | * |
| 14 | 0 | 0 | 16.5 | *** | 14 | 0 | 0 | 0 | * |
| 15 | 0 | 0 | 0 | * | 15 | 0 | 0 | 0 | * |
| 16 | 0 | 0 | 0 | * | 16 | 0 | 0 | 13.5 | *** |
| 17 | 0 | 0 | 13 | *** | 17 | 0 | 0 | 0 | * |
| 18 | 0 | 0 | 0 | * | 18 | 0 | 0 | 13 | *** |
| 19 | 20 | 19.5 | 0 | ** | 19 | 0 | 0 | 0 | * |
| 20 | 19 | 0 | 0 | ** | 20 | 0 | 0 | 16 | *** |
| Mean | 3.03 | 2.13 | 12.20 |  | Mean | 0.00 | 1.00 | 9.90 |  |
| Median | 0.00 | 0.00 | 14.75 |  | Median | 0.00 | 0.00 | 11.00 |  |
| Std Error | + 1.66 | + 1.47 | + 2.48 |  | Std Error | + 0.00 | + 1.00 | + 2.30 |  |
| 95% CI | + 3.24 | + 2.88 | + 4.86 |  | 95% CI | + 0.00 | + 1.96 | + 4.51 |  |

| Stage C-1 a | HC | | | | Stage C-1 a | TB | | | |
| --- | --- | --- | --- | --- | --- | --- | --- | --- | --- |
| Subject No. | MLCwA | MLSA-LAM | PPD | Reaction | Subject No. | MLCwA | MLSA-LAM | PPD | Reaction |
| 1 | 0 | 0 | 15.5 | *** | 1 | 14 | 8 | 20 | **** |
| 2 | 13 | 0 | 15.5 | **** | 2 | 14.5 | 0 | 15 | **** |
| **4** | 11.5 | 15.5 | 22.5 | **** | 3 | 0 | 0 | 22 | *** |
| 5 | 0 | 0 | 0 | * | 4 | 0 | 0 | 21 | *** |
| 6 | 0 | 0 | 18 | *** | 5 | 0 | 0 | 22 | *** |
| 7 | 0 | 0 | 16.5 | *** | 6 | 13 | 10 | 17 | **** |
| 8 | 11.5 | 0 | 8 | **** | 7 | 15.5 | 0 | 7.5 | **** |
| 9 | 0 | 5.5 | 11.5 | **** | 8 | 0 | 20 | 25 | **** |
| 10 | 15 | 0 | 26.5 | **** | 9 | 0 | 0 | 18.5 | *** |
| 11 | 0 | 0 | 11.5 | *** | 10 | 26.5 | 18 | 28 | **** |
| 12 | 16.5 | 15 | 16 | **** | 11 | 18.5 | 17.5 | 23 | **** |
| 13 | 17.5 | 0 | 9.5 | **** | 12 | 0 | 15.5 | 25 | **** |
| 14 | 0 | 0 | 21 | *** | 13 | 0 | 0 | 0 | * |
| 15 | 7.5 | 0 | 10.5 | **** | 14 | 0 | 0 | 17.5 | *** |
| 16 | 9.5 | 0 | 0 | ** | 15 | 10 | 0 | 18.5 | **** |
| 17 | 24 | 23.5 | 26 | **** | 16 | 10.5 | 10 | 18.5 | **** |
| 18 | 0 | 0 | 22.5 | *** | 17 | 0 | 0 | 0 | * |
| 19 | 0 | 0 | 0 | * | 18 | 9 | 0 | 24 | **** |
| 20 | 14.5 | 12.5 | 16 | **** | 19 | 0 | 0 | 16.5 | *** |
| 21 | 0 | 0 | 24 | *** | 20 | 0 | 0 | 22 | *** |
| Mean | 7.03 | 3.60 | 14.55 |  | Mean | 6.58 | 4.95 | 18.05 |  |
| Median | 3.75 | 0.00 | 15.75 |  | Median | 0.00 | 0.00 | 19.25 |  |
| Std Error | + 1.76 | + 1.58 | + 1.84 |  | Std Error | + 1.84 | + 1.66 | + 1.70 |  |
| 95% CI | + 3.46 | + 3.09 | + 3.61 |  | 95% CI | + 3.61 | + 3.25 | + 3.32 |  |

1. Phase II, Stage C-1b (Low Dose)

| Stage C-1 b | BT/TT | | | | Stage C-1 b | BL/LL | | | |
| --- | --- | --- | --- | --- | --- | --- | --- | --- | --- |
| Subject No. | MLCwA | MLSA-LAM | PPD | Reaction | Subject No. | MLCwA | MLSA-LAM | PPD | Reaction |
| 21 | 0 | 0 | 0 | * | 21 | 0 | 0 | 9.5 | *** |
| 22 | 0 | 0 | 12 | *** | 22 | 0 | 0 | 24.5 | *** |
| 23 | 0 | 0 | 15 | *** | 23 | 0 | 0 | 18 | *** |
| 24 | 0 | 0 | 17 | *** | 24 | 0 | 0 | 23 | *** |
| 25 | 0 | 0 | 0 | * | 25 | 0 | 0 | 0 | * |
| 26 | 14.5 | 10.5 | 0 | ** | 26 | 0 | 0 | 0 | * |
| 27 | 0 | 0 | 20 | *** | 27 | 0 | 0 | 19.5 | *** |
| 28 | 0 | 0 | 22.5 | *** | 28 | 0 | 0 | 10 | *** |
| 29 | 0 | 0 | 25 | *** | 29 | 0 | 0 | 0 | * |
| 30 | 0 | 0 | 7 | *** | 30 | Na | Na | Na | Na |
| 31 | 20 | 18 | 16 | *** | 31 | 0 | 0 | 0 | * |
| 32 | 0 | 0 | 20 | *** | 32 | 0 | 0 | 0 | * |
| 33 | 0 | 0 | 21 | *** | 33 | 0 | 0 | 0 | * |
| 34 | 17.5 | 12.5 | 0 | ** | 34 | 0 | 0 | 0 | * |
| 35 | 0 | 0 | 22 | *** | 35 | 0 | 0 | 0 | * |
| 36 | 11 | 15 | 18 | **** | 36 | 0 | 0 | 0 | * |
| 37 | 0 | 0 | 0 | * | 37 | 0 | 0 | 25 | *** |
| 38 | 0 | 0 | 0 | * | 38 | 0 | 0 | 0 | * |
| 39 | 0 | 0 | 0 | * | 39 | 0 | 0 | 0 | * |
| 40 | 11 | 0 | 15.5 | **** | 40 | 0 | 0 | 0 | * |
| Mean | 3.70 | 2.80 | 11.55 |  | Mean | 0.00 | 0.00 | 6.48 |  |
| Median | 0.00 | 0.00 | 15.25 |  | Median | 0.00 | 0.00 | 0.00 |  |
| Std Error | 1.53 | 1.32 | 2.13 |  | Std Error | 0.00 | 0.00 | 2.18 |  |
| 95% CI | + 2.99 | + 2.58 | + 4.16 |  | 95% CI | + 0.00 | + 0.00 | + 4.28 |  |

| Stage C-1 b | HC | | | | Stage C-1 b | TB | | | |
| --- | --- | --- | --- | --- | --- | --- | --- | --- | --- |
| Subject No. | MLCwA | MLSA-LAM | PPD | Reaction | Subject No. | MLCwA | MLSA-LAM | PPD | Reaction |
| 22 | 0 | 0 | 21 | *** | 21 | 0 | 0 | 20 | *** |
| 23 | 0 | 0 | 0 | * | 22 | 0 | 0 | 17 | *** |
| 24 | 0 | 0 | 6.5 | *** | 23 | 0 | 0 | 17 | *** |
| 25 | 0 | 0 | 26 | *** | 24 | 6 | 0 | 20.5 | **** |
| 26 | 0 | 0 | 8 | *** | 25 | 0 | 0 | 19.5 | *** |
| 27 | 0 | 0 | 0 | * | 26 | 0 | 0 | 20.5 | *** |
| 28 | 0 | 0 | 16 | *** | 27 | 0 | 0 | 19 | *** |
| 29 | 0 | 0 | 9 | *** | 28 | 0 | 0 | 20 | *** |
| 30 | 0 | 0 | 19.5 | *** | 29 | 0 | 0 | 0 | * |
| 31 | 0 | 0 | 18 | *** | 30 | 0 | 0 | 17.5 | *** |
| 32 | 0 | 0 | 9.5 | *** | 31 | 0 | 0 | 25 | *** |
| 33 | 0 | 0 | 40 | *** | 32 | 0 | 0 | 28.5 | *** |
| 34 | 10 | 11.5 | 20 | **** | 33 | 0 | 0 | 17.5 | *** |
| 35 | 0 | 0 | 15 | *** | 34 | 0 | 0 | 16.5 | *** |
| 36 | 5 | 0 | 15 | *** | 35 | 0 | 0 | 14 | *** |
| 37 | 0 | 0 | 20.5 | *** | 36 | 9 | 0 | 20 | **** |
| 38 | 0 | 0 | 0 | * | 37 | 0 | 0 | 21 | *** |
| 39 | 0 | 0 | 0 | * | 38 | 0 | 0 | 25 | *** |
| 40 | 15 | 10 | 18.5 | **** | 39 | 0 | 0 | 25 | *** |
| 41 | 16 | 0 | 18.5 | **** | 40 | 10 | 0 | 30 | **** |
| Mean | 2.30 | 1.08 | 14.05 |  | Mean | 1.25 | 0.00 | 19.68 |  |
| Median | 0.00 | 0.00 | 15.50 |  | Median | 0.00 | 0.00 | 20.00 |  |
| Std Error | + 1.15 | + 0.75 | + 2.27 |  | Std Error | + 0.70 | + 0.00 | + 1.38 |  |
| 95% CI | + 2.25 | + 1.45 | + 4.45 |  | 95% CI | + 1.37 | + 0.00 | + 2.71 |  |
